# Acupuncture modulates extracellular vesicle-miRNA-HMOX1 axis to inhibit ferroptosis in polycystic ovary syndrome: integrating machine learning, *in vitro* and *in vivo* studies

**DOI:** 10.3389/fendo.2026.1790049

**Published:** 2026-07-23

**Authors:** Xiaoling Wu, Yuanyin Li, Naxin Wang, Shumin Guo, Huiling Chen, Tingyu Chen, Liansha Huang, Hai Zhou

**Affiliations:** 1 Department of Reproductive Health, Shenzhen Bao’an Chinese Medicine Hospital, Guangzhou University of Chinese Medicine, Shenzhen, China; 2 Guangzhou University of Chinese Medicine’s Seventh Affiliated Hospital, Shenzhen, China; 3 Medical Administration Office, Shenzhen Bao’an Chinese Medicine Hospital, Guangzhou University of Chinese Medicine, Shenzhen, China

**Keywords:** acupuncture therapy, extracellular vesicles, ferroptosis, heme oxygenase-1, microRNA, polycystic ovary syndrome

## Abstract

**Background:**

Acupuncture therapy shows promise for polycystic ovary syndrome (PCOS), but its molecular mechanisms remain unclear. This study investigates whether acupuncture alleviates PCOS by regulating granulosa cell (GC) ferroptosis via extracellular vesicles (EVs).

**Methods:**

Bioinformatic analysis of KGN cell transcriptome data identified heme oxygenase-1 (HMOX1) as a key candidate. Its role was validated in a PCOS rat model and GCs using RT-qPCR, western blot, and immunohistochemistry. Ferroptosis was assessed via malondialdehyde (MDA), glutathione (GSH), and ferroptosis-related proteins (GPX4, FTH1, SLC7A11, NCOA4). HMOX1 gain/loss-of-function, rescue experiments (ferrostatin-1), and transmission electron microscopy defined its function. PCOS rats received acupuncture for 4 weeks; ovarian function and ferroptosis markers were analyzed. EVs from follicular fluid were characterized, and miRNA candidates targeting HMOX1 were screened. The EV-miRNA mechanism was tested via co-culture and inhibition assays.

**Results:**

HMOX1 was upregulated in PCOS. Its overexpression induced ferroptosis and impaired GC proliferation, reversible by ferrostatin-1. Acupuncture improved ovarian histology and oocyte maturation, concurrently normalizing HMOX1 and ferroptosis markers. It specifically enriched miR-873-5p in EVs. These EVs delivered miR-873-5p to GCs, downregulating HMOX1 and alleviating ferroptosis, an effect blocked by miR-873-5p inhibition.

**Conclusions:**

Acupuncture mitigates PCOS by promoting EV-mediated transfer of miR-873-5p, which targets HMOX1 to inhibit GC ferroptosis and restore ovarian function. This identifies a novel acupuncture-EV-miRNA-HMOX1-ferroptosis axis as a therapeutic mechanism for PCOS.

## Graphical Abstract

**Figure:**
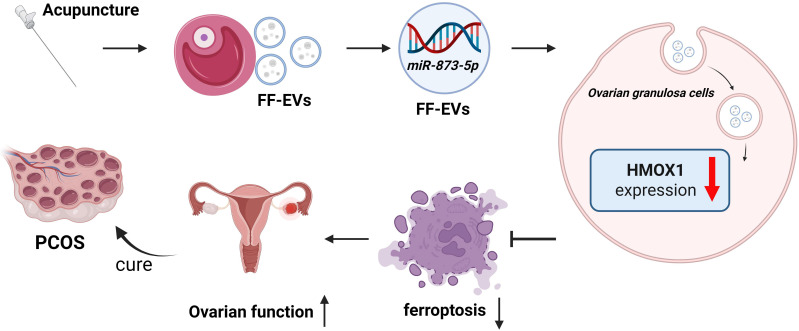
Schematic diagram of the molecular mechanism by which acupuncture therapy downregulates HMOX1 via EV-mediated miRNAs to inhibit ferroptosis in PCOS treatment.

## Introduction

Polycystic ovary syndrome (PCOS) is a prevalent endocrine and metabolic condition affecting reproductive-aged women, with an estimated global prevalence of 5%–20% (1, 2). Its characteristic clinical features include hyperandrogenemia, ovulatory dysfunction, and polycystic ovarian morphology (3). PCOS not only leads to reproductive disorders such as infertility but is also often accompanied by systemic complications, including insulin resistance, obesity, and metabolic syndrome, significantly impairing patients’ quality of life (4, 5). Although the precise pathogenesis of PCOS remains unclear, increasing evidence indicates that dysfunction of ovarian granulosa cells (GCs) may be critically involved in disease development. Recently, ferroptosis, an iron-dependent form of regulated cell death driven by lipid peroxidation, has gained considerable attention (6). It is characterized by iron-dependent lipid peroxide accumulation and collapse of the glutathione (GSH) antioxidant system (7). Notably, significant oxidative stress and abnormal lipid metabolism in the ovarian microenvironment of PCOS patients closely resemble the pathological features of ferroptosis (8, 9). However, whether ferroptosis is involved in PCOS development and its specific mechanism in GC dysfunction remains unclear. As an important component of traditional medicine, acupuncture therapy has shown unique advantages in improving reproductive endocrine function in PCOS patients (10, 11), but whether it exerts therapeutic effects by regulating ferroptosis remains to be elucidated.

Recent studies have found that key ferroptosis regulators are significantly dysregulated in ovarian tissues of PCOS patients. Among them, glutathione peroxidase 4 (GPX4) and solute carrier family 7 member 11 (SLC7A11), the most important negative regulators of ferroptosis (12), show markedly reduced expression in PCOS ovarian tissues (13, 14), suggesting that ferroptosis may contribute to PCOS pathology. Heme oxygenase-1 (HMOX1), a key enzyme in oxidative stress responses, plays a dual regulatory role in iron metabolism. Moderate activation can reduce free iron generation by degrading heme, whereas overexpression may lead to iron overload and promote ferroptosis (15, 16). Notably, HMOX1 expression is abnormally elevated in PCOS patients, but its specific molecular mechanism in GC ferroptosis requires further investigation. Therapeutically, extracellular vesicles (EVs)-mediated intercellular communication provides new perspectives for disease regulation (17, 18). Studies show that the microRNA (miRNA) expression profile in follicular fluid-derived extracellular vesicles (FF-EVs) from PCOS patients is significantly altered (19), and these changes may affect GC function by regulating downstream target genes (20). As a traditional Chinese therapy, acupuncture has unique advantages in regulating reproductive endocrine function, but research on whether it influences HMOX1 expression and ferroptosis progression by modulating the EVs-miRNA network remains completely unexplored.

This study innovatively proposes the “EVs-miRNA-HMOX1” regulatory axis hypothesis to elucidate the molecular mechanisms of acupuncture in treating PCOS. As natural nanoscale vesicles, EVs can stably transport nucleic acid molecules, such as miRNAs, through bodily fluids to facilitate intercellular communication. Proteomic analysis reveals significant alterations in protein composition, particularly iron metabolism-related proteins, within FF-EVs from PCOS patients. Furthermore, serum exosomal miRNA profiling demonstrates characteristic changes in PCOS, with miR-873-5p (microRNA-873-5p) expression levels showing a negative correlation with disease severity. Bioinformatics predictions indicate that miR-873-5p may directly bind to the 3’ untranslated region (3’UTR) of HMOX1 mRNA. Clinical observations reveal a significant increase in miRNA cargo in FF-EVs following acupuncture treatment. This study comprehensively clarifies how HMOX1 regulates ferroptosis in granulosa cells during PCOS, reveals the influence of acupuncture on EV-derived miRNA profiles, and highlights the miR-873-5p/HMOX1 axis as a key mechanism underlying acupuncture-mediated therapeutic effects. These results offer theoretical support for the development of EV-based noninvasive diagnostic approaches and targeted treatment strategies.

The current research aims to clarify the precise molecular mechanisms by which acupuncture therapy inhibits GC ferroptosis in PCOS through the EVs-miRNA-HMOX1 signaling axis. We systematically analyze how acupuncture-induced changes in EVs miRNA regulate HMOX1 expression and evaluate their therapeutic value in improving ovarian function in PCOS. These discoveries not only deepen our understanding of PCOS pathogenesis but also provide theoretical support for developing novel, EV-based, non-invasive treatment strategies. However, individual variations in acupuncture protocols may affect study reproducibility, and the delivery efficiency and targeting specificity of EVs-miRNA *in vivo* require further optimization. Future studies should focus on standardizing acupuncture parameters and improving the isolation and purification techniques for EVs to enhance the translational potential of acupuncture. Subsequent research could investigate how various acupuncture regimens affect the miRNA profiles of EVs and develop miRNA-specific targeted delivery systems, thereby opening new avenues for precision medicine in PCOS treatment.

## Materials and methods

### High-throughput sequencing and analysis of KGN cells

High-throughput sequencing and subsequent analysis were conducted on KGN cells. A total of ten samples, including three from the control group and seven from the PCOS model group, were randomly chosen. Total RNA was isolated using a commercial RNA extraction kit (Invitrogen, USA) and quantified based on optical density measurements obtained with a UV–visible spectrophotometer (Eppendorf, USA). RNA quality and integrity were further verified by agarose gel electrophoresis. Qualified RNA samples were then reverse-transcribed into complementary DNA (cDNA) for library preparation. Sequencing was carried out on the Illumina NextSeq 500 platform, and raw image files were processed into sequencing reads via base calling. To improve data quality, adaptor sequences and low-quality reads were removed using Cutadapt, yielding clean reads for downstream analysis. These reads were subsequently aligned to the human reference genome with HISAT2, and gene expression levels were quantified using R-based tools to construct an expression matrix.

### Screening of differentially expressed genes

The R package “limma” was used to identify DEGs from the high-throughput sequencing data, with |log_2_ FC| > 2 and *p*.adjust < 0.01 set as the thresholds. Volcano plots and heatmaps were produced with the R packages “ggplot2” and “pheatmap”, respectively. Gene ontology (GO) and Kyoto Encyclopedia of Genes and Genomes (KEGG) pathway enrichment analyses of the DEGs were conducted via the Sangerbox database (http://sangerbox.com/).

### Machine learning-based screening of key gene sets

Three machine learning algorithms—least absolute shrinkage and selection operator (LASSO), support vector machine (SVM), and random forest (RF)—were employed to identify key gene sets. LASSO regression analysis was performed using the R package glmnet (v4.0-2), SVM analysis using e1071 (v1.7-3), and RF analysis using randomForest (v4.6-14). The dataset was partitioned into training (70%) and test (30%) sets.

### miRNA prediction analysis

Upstream miRNAs regulating HMOX1 were predicted using three databases: TargetScanHuman (v8.0), miRDB, and StarBase (v2.0). The gene symbol “HMOX1” was queried with the species set to human, and only conserved results were retained from TargetScanHuman. Predicted miRNAs from all databases were consolidated and intersected using the jvenn online tool. The overlapping miRNAs were selected as candidates and tabulated for subsequent functional validation.

### KGN cell culture and PCOS modeling

The human ovarian GC line KGN was obtained from the China Center for Type Culture Collection (catalog number: GDC0707, China). Cells were cultured in DMEM/F12 medium (11330032, Gibco, USA) supplemented with 10% fetal bovine serum (FBS, A5670701, Gibco, USA) and 1% penicillin-streptomycin (15140122, Gibco, USA) at 37 °C with 5% CO_2_ in a humidified incubator (Thermo Scientific, USA). To establish the PCOS model, KGN cells were treated with high-concentration insulin (100 nM, I9278, Sigma-Aldrich, USA) and dihydrotestosterone (10 nM, D-073, Sigma-Aldrich, USA) for 48 hours.

The experimental groups were designated as follows: Control, PCOS model group, lentiviral negative control group (NC-OE), HMOX1 overexpression (HMOX1-OE) group, HMOX1-OE plus ferroptosis inhibitor ferrostatin-1 treatment group (HMOX1-OE+Fer-1), miRNA mimic negative control, miR-873-5p mimic transfection group (miR-873-5p mimic), PCOS plus inhibitor NC group (PCOS+inhibitor NC), acupuncture treatment plus inhibitor NC group (Acupuncture, ACU+NC), and acupuncture treatment plus miR-873-5p inhibitor group (ACU+miR-873-5p inhibitor). For the HMOX1-OE+Fer-1 group, cells were treated with 10 μM ferrostatin-1 (Fer-1) (HY-100579, MedChemExpress, USA) for 24 hours.

### Lentiviral overexpression cell line construction

Lentiviral vectors carrying HMOX1-OE or empty vector (NC-OE) were constructed using the GV492 backbone (Shanghai GeneChem Co., China). KGN cells were infected at a multiplicity of infection of 30, followed by puromycin (2 μg/mL, P8833, Sigma-Aldrich, USA) selection for 7 days to establish stable lines. Infection efficiency was validated by reverse transcription-quantitative polymerase chain reaction (RT-qPCR) and Western blot (WB).

### miRNA mimic and inhibitor transfection

The synthetic miRNA mimic (miR-873-5p mimic) and inhibitor (miR-873-5p inhibitor) were provided by RiboBio (Guangzhou, China). Transfection was performed using Lipofectamine 2000 transfection reagent (11668019, Invitrogen, USA). After 20 minutes of incubation at room temperature, the transfection complex was added to the cells for 48 hours before harvesting for quantitative polymerase chain reaction and protein analysis.

### Cell Counting Kit-8 cell viability assay

Cells (5×10^3^/well) were seeded in 96-well plates. After 24-hour treatments, 10 μL CCK-8 reagent (CK04, Dojindo, Japan) was added, followed by 2-hour incubation at 37 °C. Absorbance was measured at 450 nm using a microplate reader (BioTek Synergy H1, USA). Each group included six technical replicates, and the assay was repeated in three independent experiments.

### 5-ethynyl-2’-deoxyuridine proliferation assay

Cells were plated in 24-well plates and exposed to 10 μM EdU (C10310-1, RiboBio, China) for 2 hours at 37 °C to assess proliferative activity. After fixation, nuclei were stained with Apollo488 and counterstained with 4’,6-diamidino-2-phenylindole. Images were acquired using a fluorescence microscope (Leica DMi8, Germany), with five random fields per group quantified. EdU-positive rates were calculated using ImageJ.

### Transmission electron microscopy observation

Treated KGN cells were fixed overnight with 2.5% glutaraldehyde (G5882, Sigma-Aldrich, USA), dehydrated through an ethanol gradient, and embedded in epoxy resin. Ultrathin sections were prepared and stained with uranyl acetate (22400, EMS, USA) and lead citrate (22440, EMS, USA). Mitochondrial morphology and ultrastructure were examined using a TEM (Hitachi HT7700, Japan).

### Determination of Fe^2+^ content

Levels of intracellular and tumor-associated iron were quantified using a commercial iron detection kit (ab83366, Abcam, UK) in accordance with the manufacturer’s protocol (21).

### Dual-luciferase reporter assay

HEK-293T cells (CRL-11268, ATCC, USA) were co-transfected with pGL3 reporter vectors carrying either wild-type or mutant HMOX1 3′UTR sequences together with miR-873-5p mimic, using Lipofectamine 3000 (L3000015, Invitrogen, USA). After 48 hours, firefly and Renilla luciferase activities were detected with the Dual-Luciferase Reporter Assay System (E1910, Promega, USA) on a GloMax multimode microplate reader (Promega, USA). Results were normalized as relative luciferase units.

### FF-EVs isolation and characterization

Follicular fluid was collected from PCOS model and acupuncture-treated rats (n=6 per group). Sequential centrifugation was performed at 4 °C (300 × g for 10 min, 2,000 × g for 20 min, 10,000 × g for 30 min) to remove cellular debris, followed by ultracentrifugation at 100,000 × g (Beckman Coulter Optima XPN-100, USA) to isolate EVs. The EVs were resuspended and characterized by TEM (JEM-1400, Japan) for morphological analysis and nanoparticle tracking analysis (NTA) (NanoSight NS300, Malvern, UK) for size distribution. EV markers were verified by WB using antibodies against CD81 (ab59479, Abcam, USA), TSG101 (ab125011, Abcam, USA), and ALIX (#2171, Cell Signaling Technology, USA).

### PCOS rat model establishment and grouping

Female Sprague-Dawley rats (6–8 weeks old, 180–220 g) were purchased from Beijing Vital River Laboratory Animal Technology Co., Ltd. All animals were housed under specific pathogen-free conditions (temperature 22 ± 2 °C, humidity 50% ± 10%, 12-hour light/dark cycle) with free access to food and water. After a 7-day acclimatization period, the PCOS model was established via daily intraperitoneal injection of dehydroepiandrosterone (DHEA; 252805, Sigma-Aldrich, USA) at a dose of 60 mg/kg dissolved in 0.2 mL sesame oil for 21 consecutive days, while control rats received sesame oil alone. After model establishment, animals were randomly assigned to the following groups: Control, PCOS, acupuncture treatment (ACU), acupuncture plus HMOX1 inhibitor SnPP (ACU+SnPP), and acupuncture plus erastin treatment (ACU+Erastin). The ACU+Erastin group received erastin (80 mg/kg/day, i.p.) once daily for 3 consecutive days before PCOS induction (22). The SnPP group received SnPP (sc-203452A, Santa Cruz Biotechnology, Dallas, TX, USA) at 10 mg/kg by intragastric administration once daily for 3 consecutive days before PCOS induction (23).

### Acupuncture treatment

During acupuncture treatment, rats were placed in specialized rat restrainers in a supine position with their trunks immobilized while allowing free movement of their heads and limbs. According to the Experimental Animal Acupuncture Atlas compiled by the Experimental Acupuncture Branch of the Chinese Acupuncture Society, the acupoints selected included “Guanyuan” (approximately 25 mm below the umbilicus), “Zigong” (approximately 30 mm below the umbilicus), and “Sanyinjiao” (30 mm below the umbilicus and 10 mm proximal to the medial malleolus). The Conception Vessel acupoints “Zhongji,” “Guanyuan,” and “Qihai” were localized on rats by proportionally translating human anatomical measurements to 34 mm, 25 mm, and 12.5 mm below the umbilicus (equivalent to 4, 3, and 1.5 cun in humans, respectively). Sterile disposable acupuncture needles (0.25 × 25 mm, Huatuo Brand, China) were vertically inserted into these acupoints to a depth of 5–8 mm, with needle sensation achieved through gentle manipulation to induce slight muscle twitching. Treatments were administered for 30 minutes daily over 15 consecutive days. For the sham acupuncture group, mice underwent identical restraint procedures, but needles were superficially inserted into non-acupoint regions (5 mm lateral to “Guanyuan,” “Zhongji,” “Qihai,” “Sanyinjiao,” and “Zigong”) at the same depth of 5–8 mm with equivalent needle manipulation, also administered for 30 minutes daily over 15 days.

### Hematoxylin and eosin staining

Ovarian and uterine tissues were fixed in 10% neutral buffered formalin (G2161, Solarbio, China) for 24 hours, followed by dehydration, embedding, and sectioning at a thickness of 5 μm. After H&E staining (C0105, Beyotime, China), slides were observed under a light microscope (Leica DM2500, Germany). Tissue morphology was analyzed using ImageJ (v1.53).

### *In vitro* maturation of oocytes

Ovaries were aseptically isolated from each group of rats, and cumulus-oocyte complexes (COCs) were collected from the follicular fluid. COCs were cultured in M199 medium (11150059, Thermo Fisher Scientific, USA) supplemented with 10% FBS (10099141, Gibco, USA), 0.075 IU/mL luteinizing hormone (LH; YL2501015, Shanghai YuanMu Biological Technology Co. Ltd., China), and 0.075 IU/mL follicle-stimulating hormone (F4021, Sigma-Aldrich, USA) at 37 °C with 5% CO_2_ for 24 hours. Cumulus expansion and polar body extrusion rates were observed under a microscope (Nikon Eclipse Ti2, Japan) (13).

### Lipid peroxidation and antioxidant indicators

After decapitation, serum and ovarian tissue homogenates were collected from rats in each group for the measurement of malondialdehyde (MDA), an end product of lipid peroxidation, and glutathione (GSH), an antioxidant indicator. Cell culture supernatants were also collected for analysis. MDA and GSH levels were determined using commercial assay kits (A003–1 and A006-1, Nanjing Jiancheng Bioengineering Institute, China), and absorbance was measured using a microplate reader (BioTek Synergy H1, USA). Six animals were included in each group, and the data were used for subsequent statistical ana.

### Localization of HMOX1 by immunohistochemistry

Ovarian paraffin sections were dewaxed, rehydrated, and subjected to antigen retrieval (Tris-EDTA, pH 9.0). Endogenous peroxidase was blocked with 3% H_2_O_2_, followed by blocking with 10% goat serum (SL038, Solarbio, China). Sections were incubated overnight at 4 °C with primary antibody against HMOX1 (ab13243, Abcam, USA; 1:200 dilution), followed by horseradish peroxidase (HRP)-conjugated secondary antibody (ZB-2301, ZSGB-BIO, China). Diaminobenzidine was used for chromogenic development, with hematoxylin counterstaining. After dehydration, sections were mounted and observed under a light microscope (Leica DM2500, Germany) to localize HMOX1 expression in GC.

### Isolation and culture of GC

Ovarian tissues from PCOS model and control rats were aseptically dissected to isolate follicles. Follicular fluid was filtered through a 75 μm mesh, and cells were collected by centrifugation at 1,000 rpm for 5 minutes. The cell pellet was resuspended in DMEM/F12 medium (11330032, Gibco, USA) supplemented with 10% FBS and seeded into 6-well plates. Cells were maintained at 37 °C with 5% CO_2_. After 24 hours of adherence, the medium was replaced with fresh culture medium, and cells were harvested for RNA and protein extraction.

### Gene expression analysis by RT-qPCR

Total RNA was isolated from tissues or cultured cells using TRIzol reagent (15596026, Invitrogen, USA). RNA concentration and purity were determined by measuring the absorbance ratio at 260/280 nm with a NanoDrop Lite spectrophotometer (ND-LITE-PR, Thermo Scientific, Germany). Complementary DNA (cDNA) was generated from total RNA using the PrimeScript RT reagent kit with gDNA Eraser (RR047Q, TaKaRa, Japan). Quantitative PCR was subsequently carried out using SYBR Green PCR Master Mix (4364344, Applied Biosystems, USA) on an ABI PRISM 7500 Sequence Detection System (Applied Biosystems, USA).

Gene-specific primers were synthesized by TaKaRa (**Supplementary Table 1**), with GAPDH serving as the internal control gene. Relative gene expression levels were calculated using the 2^-ΔΔCt^ method. All RT-qPCR experiments were performed in triplicate.

### Protein expression detection by WB

Protein samples were prepared from tissues or cells using protease inhibitor-containing RIPA buffer (P0013B, Beyotime, China). After quantification with a BCA assay kit (P0012, Beyotime, China), equal quantities of protein were resolved on 10% SDS-PAGE gels and electrotransferred to PVDF membranes (FFP39, Beyotime, China). The membranes were blocked with 5% BSA for 2 hours and then probed with the indicated primary antibodies against human or rat proteins (**Supplementary Table 2**), followed by incubation with HRP-labeled goat anti-rabbit IgG (ab6721, 1:2000; Abcam, UK). Pierce ECL WBting Substrate (32209, Thermo Scientific, Germany) was prepared by mixing equal volumes of Solutions A and B in the dark, then applied to the membranes. WB signals were visualized using a Bio-Rad imaging system (Bio-Rad, USA). Band intensities were analyzed with ImageJ software and normalized to β-actin. All assays were independently conducted three times.

### Statistical analysis

All experiments were conducted in triplicate, and results are expressed as mean ± standard deviation (SD). Data distribution and variance homogeneity were evaluated using the Shapiro–Wilk test and Levene’s test, respectively. For normally distributed data with equal variances, comparisons between two groups were performed using a two-tailed Student’s *t*-test, while multiple-group comparisons were analyzed by one-way ANOVA followed by Tukey’s *post hoc* test. When variance homogeneity was not met, Welch’s t-test was applied for two-group comparisons, and Brown–Forsythe ANOVA with Dunnett’s T3 *post hoc* test was used for multiple groups. Statistical analyses were carried out using GraphPad Prism 9.0 (GraphPad Software, USA), with *p* < 0.05 considered statistically significant.

## Results

### Bioinformatics analysis reveals significant upregulation of HMOX1 in PCOS

**Figure 1:**
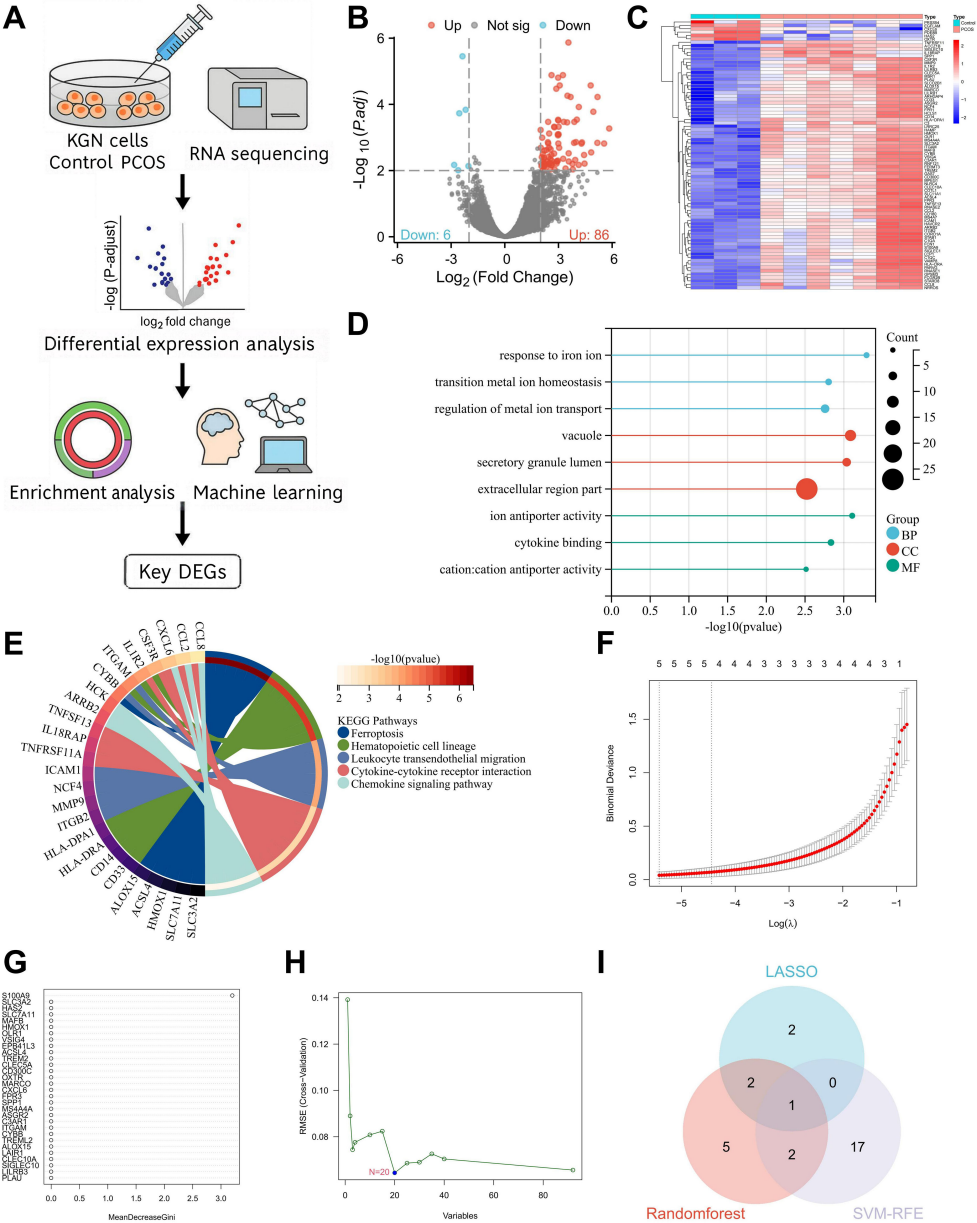
Screening of HMOX1 as a key DEGs in PCOS. **(A)** Bioinformatics workflow showing transcriptome sequencing, differential gene screening, enrichment analysis, and machine learning algorithm selection steps; **(B)** Volcano plot of differentially expressed mRNAs between Control (n=3) and PCOS (n=3) groups in KGN cells from high-throughput sequencing data (blue dots: significantly downregulated mRNAs; red dots: significantly upregulated mRNAs; gray dots: non-significant mRNAs); **(C)** Heatmap demonstrating differential gene expression patterns in Control and PCOS KGN cell samples; **(D)** GO enrichment analysis of upregulated genes, showing major classifications in BP, cellular components, and molecular functions; **(E)** KEGG pathway enrichment analysis of upregulated genes; **(F)** LASSO coefficient screening plot; **(G)** RF algorithm results; **(H)** SVM-RFE analysis results; **(I)** The Venn diagram illustrates the intersection of disease-related factors screened by three machine learning algorithms: LASSO regression, RF algorithm, and SVM-RFE. In the volcano plot, blue dots represent significantly downregulated mRNAs in KGN cells, red dots indicate significantly upregulated mRNAs in KGN cells, and gray dots denote mRNAs with no significant differential expression.

The human ovarian GC line KGN was cultured and stimulated to establish a PCOS model. Transcriptome sequencing was performed on KGN cells from both Control and PCOS group (**Figure 1A**). Using the R package limma, 92 DEGs were screened, including 86 upregulated and 6 downregulated genes (**Figures 1B, C**). GO and KEGG enrichment analyses revealed significant alterations in gene expression profiles in PCOS-modeled KGN cells. Upregulated genes were predominantly enriched in biological processes (BP), such as “iron ion response” and “regulation of metal ion transport” (**Figure 1D**). KEGG results further showed enrichment in ferroptosis-related pathways (**Figure 1E**).

Further multivariate Cox regression analysis using LASSO (**Figure 1F**), support vector machine-recursive feature elimination (SVM-RFE) (**Figure 1G**), and RF algorithms (**Figure 1H**) identified HMOX1 as the most critical mRNA (**Figure 1I**). These results indicate that HMOX1 may participate in PCOS development by modulating GC ferroptosis.

### Ferroptosis occurrence and phenotypic validation in PCOS model

**Figure 2:**
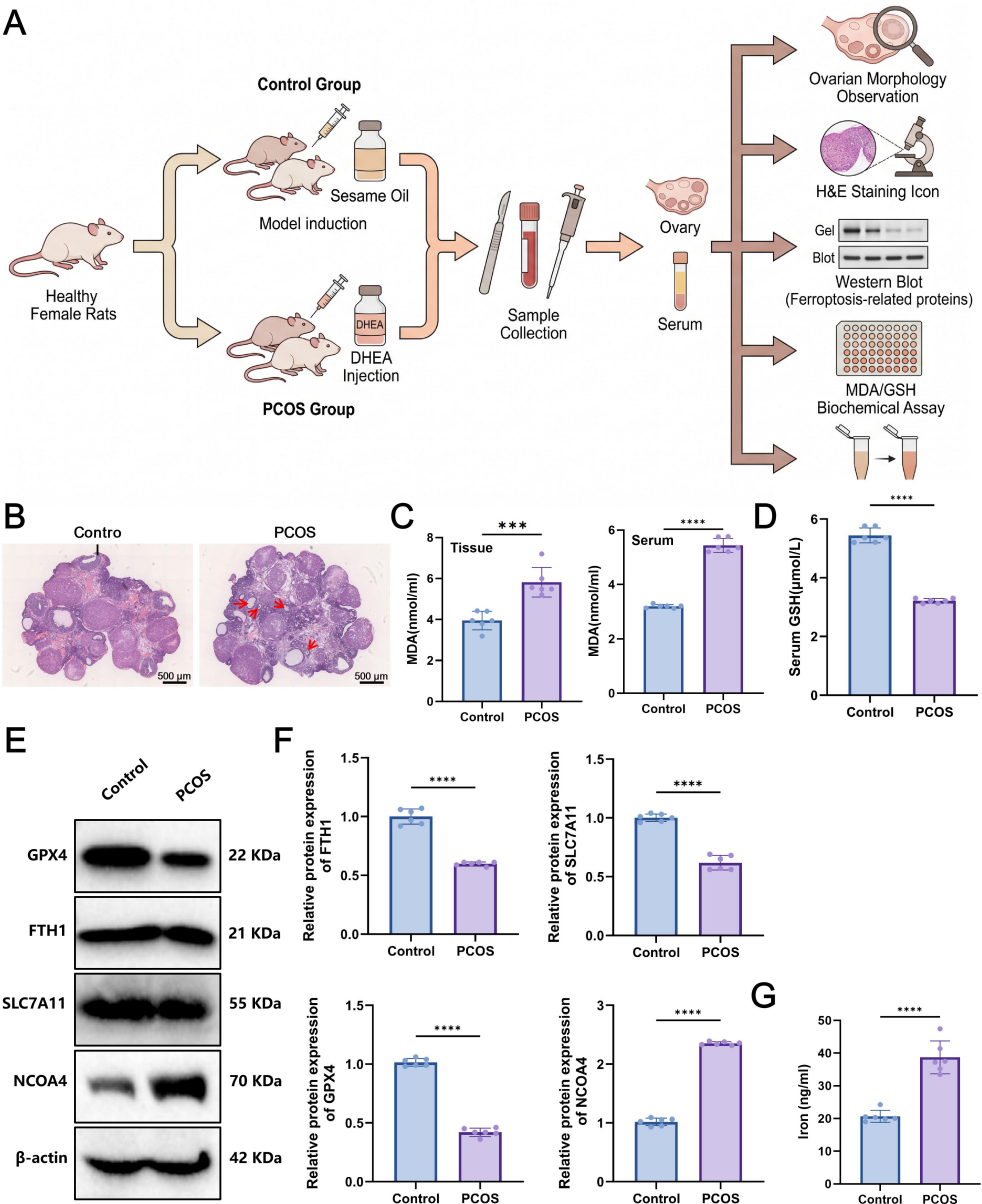
Occurrence and phenotypic validation of ferroptosis in PCOS model. **(A)** Experimental flowchart showing PCOS model establishment, histological evaluation, and ferroptosis-related detection procedures; **(B)** H&E staining showing ovarian tissue morphology in control and PCOS model groups (scale bar: 500 µm); **(C, D)** Measurements of MDA and GSH levels in serum and ovarian tissues; **(E, F)** WB analysis of ferroptosis markers (GPX4, FTH1, SLC7A11) and the ferritinophagy marker NCOA4 in ovarian tissues; **(G)** Kit-based measurement of ovarian iron levels in each group. Animal experiments included 6 rats per group. ***p < 0.001, ****p < 0.0001 for intergroup comparisons.

To further investigate the involvement of GC ferroptosis in the development of PCOS, rats were randomly assigned to either a control group or a PCOS model group. The PCOS rat model was established through consecutive drug administration to confirm the occurrence of ferroptosis (**Figure 2A**). After the intervention period, ovarian and uterine tissues were collected for morphological and functional evaluation. Compared with controls, PCOS rats exhibited significantly enlarged ovaries with hardened texture and atrophic uteri. H&E staining revealed markedly increased cystic follicles, reduced corpus luteum formation, and disordered follicular development in PCOS ovaries (**Figure 2B**), confirming successful model establishment.

To further assess the involvement of ferroptosis in ovarian pathology, serum and ovarian tissue samples were analyzed for lipid peroxidation and antioxidant indicators. Results show that PCOS rats exhibited significantly elevated MDA levels and reduced GSH levels in both serum and ovarian tissues compared to controls (**Figures 2C, D**), indicating enhanced lipid peroxidation and impaired GSH-dependent antioxidant capacity.

WB analysis of ferroptosis markers demonstrated significantly downregulated expression of GPX4, ferritin heavy chain 1 (FTH1), and SLC7A11, alongside significantly upregulated nuclear receptor coactivator 4 (NCOA4), in the PCOS group (**Figures 2E, F**). In addition, iron assay results showed a marked elevation of ovarian iron content in PCOS rats compared with controls (**Figure 2G**). Collectively, these results demonstrate activated ferroptosis in PCOS model rats, suggesting its potential involvement in ovarian dysfunction.

### Significant upregulation of HMOX1 in PCOS ovarian tissues

**Figure 3:**
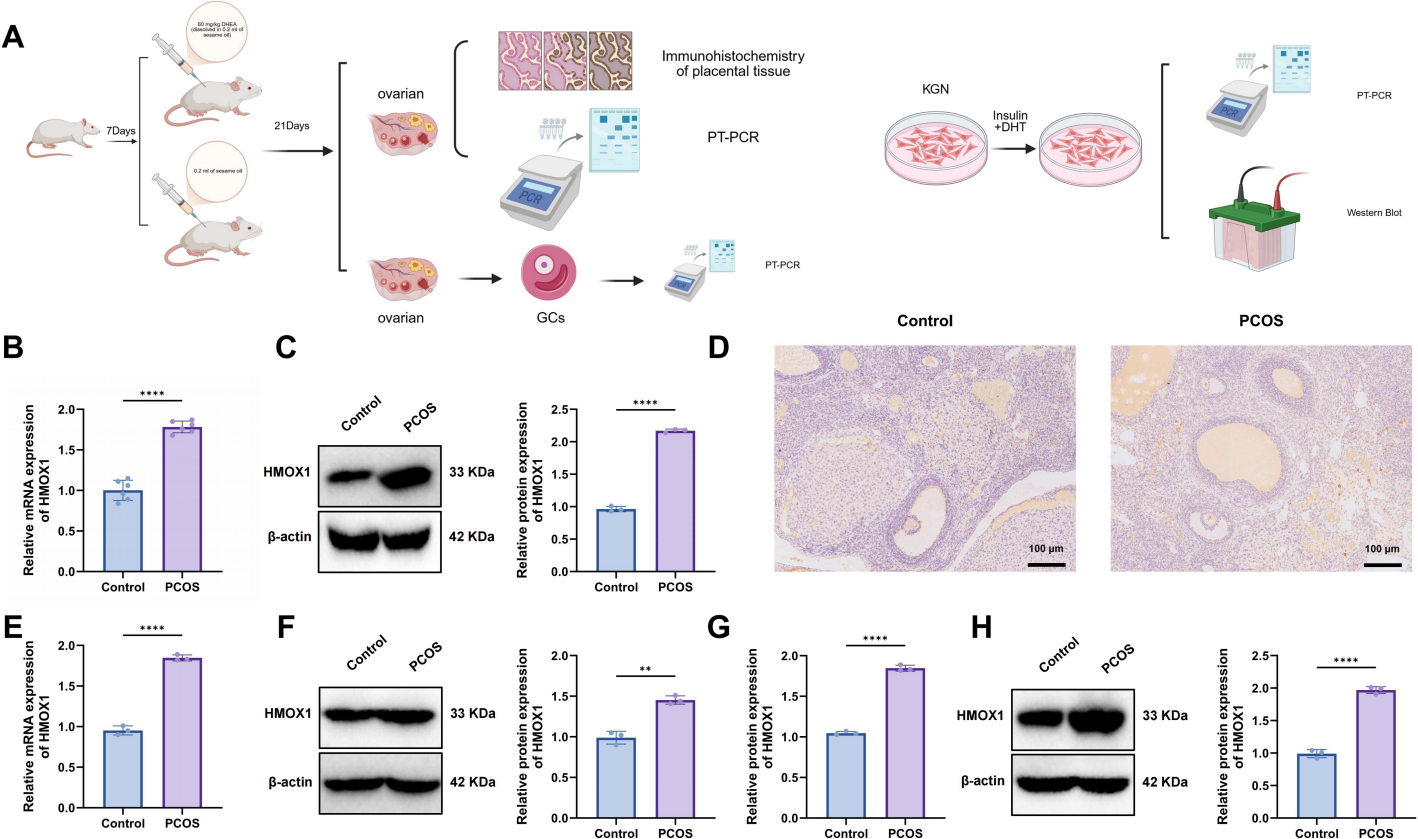
Validation of HMOX1 expression at tissue and cellular levels in the PCOS model. **(A)** Experimental flowchart illustrating PCOS rat model establishment, ovarian tissue and GC isolation, and HMOX1 expression detection; **(B)** RT-qPCR analysis of HMOX1 mRNA expression in rat ovarian tissues; **(C)** WB detection of HMOX1 protein expression in ovarian tissues; **(D)** IHC staining showing HMOX1 localization and expression in rat ovarian tissues (bar: 100 μm); **(E)** RT-qPCR measurement of HMOX1 mRNA expression in GCs from normal and PCOS rats; **(F)** WB analysis of HMOX1 protein expression in GCs; **(G)** RT-qPCR detection of HMOX1 mRNA expression in KGN cells under PCOS-mimicking conditions; **(H)** WB analysis of HMOX1 protein expression in KGN cells. All cellular experiments were performed in triplicate. **p < 0.01, ****p < 0.0001 for intergroup comparisons.

We established a PCOS rat model and conducted multi-level experiments to validate the expression patterns of HMOX1 (**Figure 3A**). Rats were divided into control and PCOS, with ovarian tissues collected post-modeling for molecular analysis. RT-qPCR revealed significantly elevated HMOX1 mRNA expression in PCOS ovarian tissues versus controls (**Figure 3B**). WB analysis confirmed concurrent upregulation of HMOX1 protein. Similarly, HMOX1 expression was significantly increased in the PCOS model group (**Figure 3C**). IHC demonstrated predominant HMOX1 localization in GC of follicular structures, with significantly enhanced HMOX1-positive signals in PCOS samples (**Figure 3D**).

At the cellular level, we isolated and cultured ovarian GC from PCOS model rats and used normal GS as a control to examine changes in HMOX1 expression. RT-qPCR analysis revealed a significant increase in HMOX1 mRNA levels in PCOS GC compared with normal controls (**Figure 3E**). Consistently, Western blot results confirmed elevated HMOX1 protein expression in PCOS GC relative to the control group (**Figure 3F**).

These findings were further corroborated in the human KGN cell line, where PCOS-mimicking treatment significantly upregulated HMOX1 mRNA and protein expression compared to untreated cells (**Figures 3G, H**). Collectively, these results demonstrate consistent HMOX1 upregulation across species and experimental systems, establishing a foundation for mechanistic investigations.

### HMOX1 inhibits GC proliferation by inducing ferroptosis

**Figure 4:**
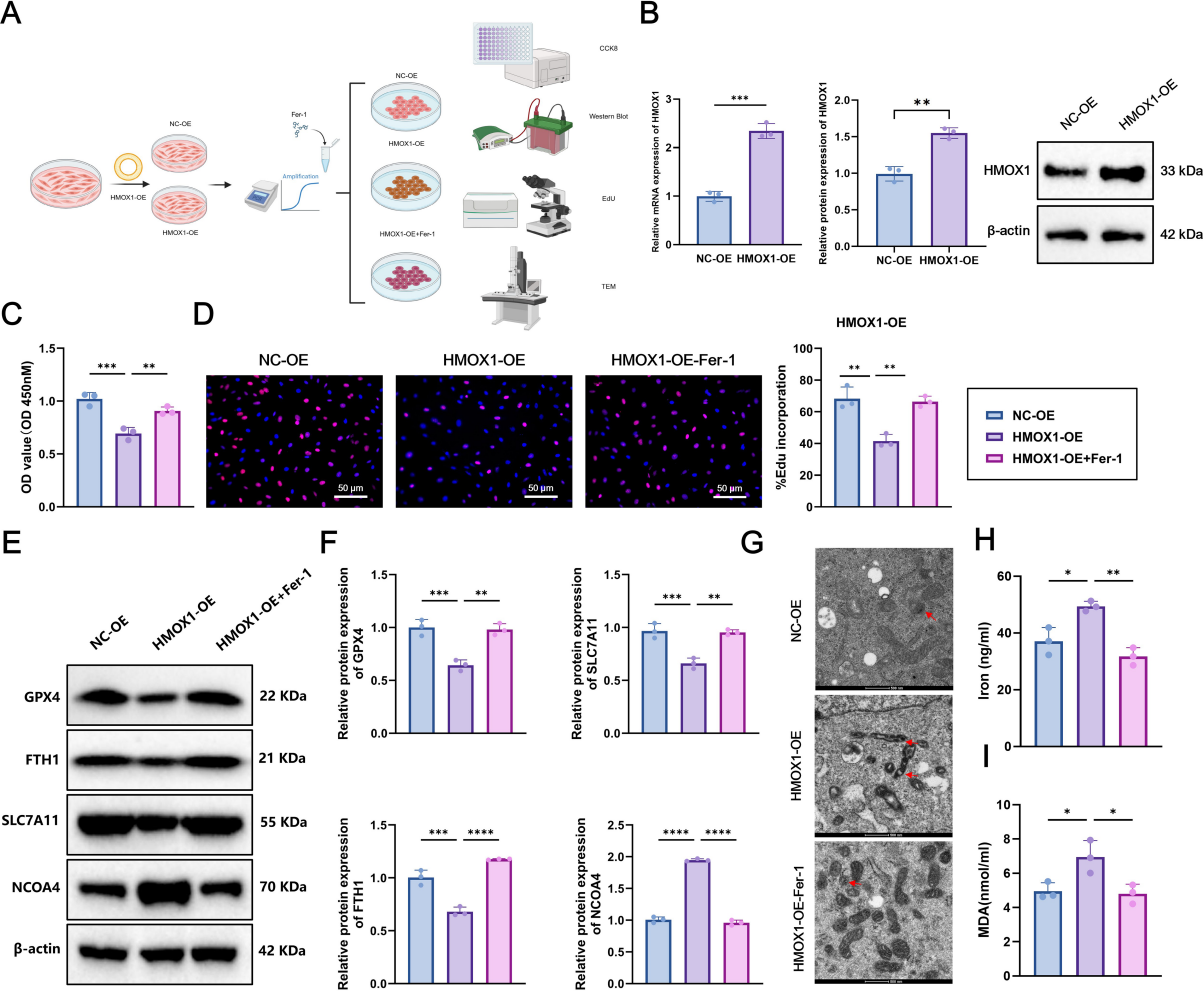
Mechanistic validation of HMOX1-mediated ferroptosis in inhibiting KGN cell proliferation. **(A)** Experimental flowchart illustrating the three-group treatment protocol and detection procedures; **(B)** RT-qPCR and Western blot analysis of HMOX1 expression levels across groups; **(C)** CCK-8 assay measuring cell viability changes; **(D)** EdU assay evaluating cell proliferation capacity (bar: 50 μm); **(E, F)** WB detection of GPX4, FTH1, SLC7A11 and NCOA4 expression in different groups; **(G)** TEM images showing mitochondrial morphology in KGN cells across groups (bar: 500 nm); **(H)** Intracellular iron levels in each group measured using a commercial assay kit; **(I)** Changes in MDA levels in each group. All cellular experiments were performed in triplicate. *p < 0.05, **p < 0.01, ***p < 0.001, and ****p < 0.0001 indicate statistically significant differences between groups.

We established three experimental groups: NC-OE group, HMOX1-OE group, and HMOX1-OE+Fer-1 group to evaluate the effects of HMOX1 on GC ferroptosis and proliferation, with rescue experiments investigating the reversible mechanism (**Figure 4A**). Stable HMOX1-OE KGN cell lines were constructed, WB and RT-qPCR verified overexpression efficiency. Compared with the NC-OE group, the HMOX1-OE group showed markedly increased HMOX1 mRNA and protein levels, indicating successful construction of the overexpression model (**Figure 4B**).

Functional assays demonstrated that CCK-8 results showed significantly decreased cell viability in the HMOX1-OE group compared to NC-OE group, while the HMOX1-OE+Fer-1 group exhibited significantly increased viability relative to HMOX1-OE (**Figure 4C**). EdU assays revealed significantly reduced EdU-positive rates in HMOX1-OE cells versus NC-OE group, indicating impaired proliferation capacity, whereas the HMOX1-OE+Fer-1 group showed marked recovery of EdU-positive rates (**Figure 4D**).

To further validate whether HMOX1 functions through the ferroptosis pathway, we examined the expression of related proteins. WB results demonstrated significantly downregulated expression of ferroptosis-inhibitory proteins GPX4, FTH1 and SLC7A11, along with significantly upregulated iron autophagy-related protein NCOA4 in HMOX1-OE versus NC-OE group. In contrast, the HMOX1-OE+Fer-1 group showed significantly increased GPX4, FTH1 and SLC7A11 expression and decreased NCOA4 expression compared to HMOX1-OE (**Figures 4E, F**).

Finally, TEM observation of cellular ultrastructure revealed that compared with the NC-OE group, HMOX1-OE cells exhibited markedly shrunken mitochondria, disappeared cristae, and increased membrane density, which are characteristic features of ferroptosis, whereas these morphological alterations were substantially alleviated in the HMOX1-OE+Fer-1 group (**Figure 4G**). Biochemical assays further revealed that intracellular iron content was markedly elevated in the HMOX1-OE group relative to NC-OE, whereas this increase was significantly attenuated following Fer-1 treatment (HMOX1-OE+Fer-1) (**Figure 4H**). In addition, MDA levels were significantly increased in the HMOX1-OE group and significantly decreased after Fer-1 treatment (**Figure 4I**). Together, these results demonstrate that HMOX1 inhibits GC proliferation by inducing ferroptosis, an effect that can be significantly reversed by the ferroptosis inhibitor Fer-1.

### Acupuncture alleviates ovarian dysfunction and improves oocyte maturation by suppressing ferroptosis

**Figure 5:**
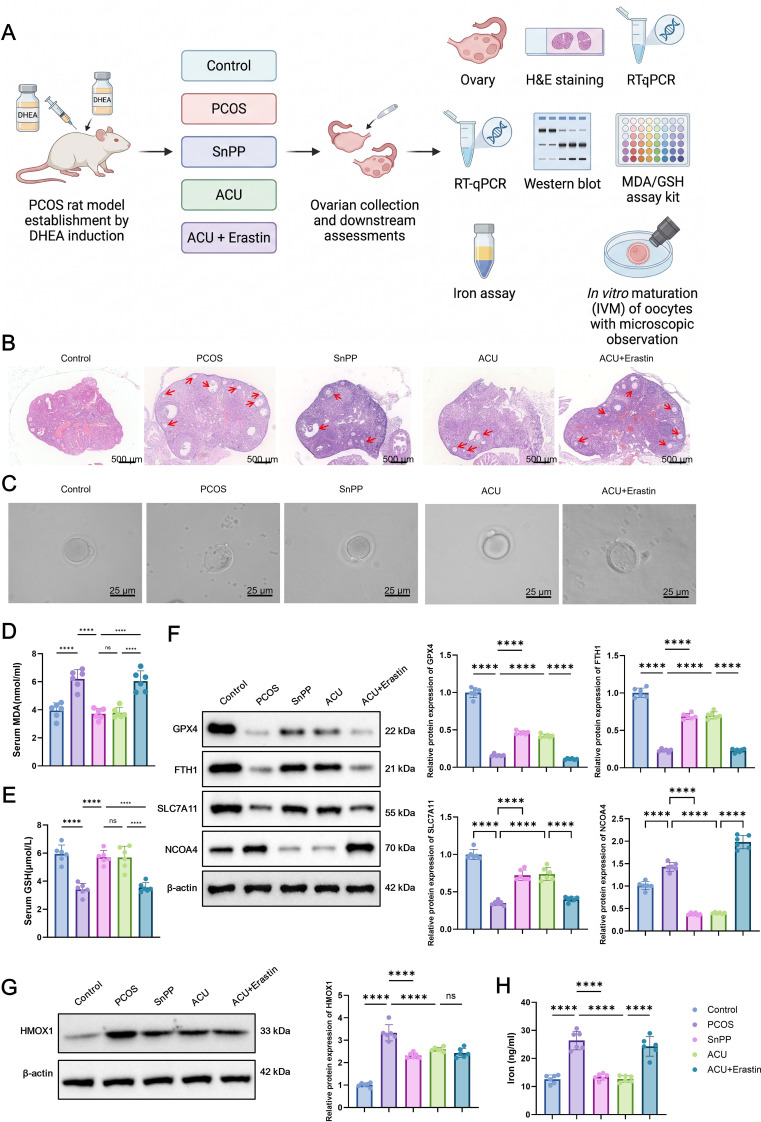
Acupuncture ameliorates ovarian dysfunction and suppresses ferroptosis in PCOS. **(A)** Experimental flowchart illustrating the three-group treatment strategy, acupuncture intervention, and related detection procedures; **(B)** H&E staining showing ovarian tissue morphology across groups (bar: 500 μm); **(C)** IVM culture demonstrating oocyte polar body extrusion and cumulus expansion (bar: 25 μm); **(D, E)** Measurements of MDA and GSH levels in serum and ovarian tissues; **(F, G)** WB analysis of GPX4, FTH1, SLC7A11, NCOA4 and HMOX1 protein expression; **(H)** Ovarian iron levels in each group measured using a commercial assay kit. Animal experiments included 6 mice per group. **** indicates *p* < 0.0001 for intergroup comparisons; ns denotes no significant difference.

We established a PCOS rat model and randomly divided the animals into four groups: the PCOS model group (PCOS), the PCOS + Hmox1 inhibitor group (SnPP), the acupuncture treatment group (Acupuncture, ACU), and the acupuncture treatment plus ferroptosis activator group (ACU+Erastin), in order to validate the therapeutic effect of acupuncture in PCOS and its mechanism in regulating ferroptosis (**Figure 5A**). The intervention lasted for 4 consecutive weeks. At the end of the treatment period, ovarian and uterine tissues were collected from each group to evaluate ovarian appearance, texture, ovulatory function, and histological changes. Compared with the PCOS group, the ovaries in the ACU and SnPP groups were smaller and softer in texture. H&E staining showed that follicular development was improved in the ACU group, with fewer cystic follicles and more corpora lutea (**Figure 5B**). Compared with the ACU group, the ACU+Erastin group exhibited an increased number of cystic follicles and fewer corpora lutea, suggesting that the therapeutic effect of acupuncture may be mainly mediated through the regulation of ferroptosis.

To further assess the effect of acupuncture on oocyte maturation, immature oocytes were collected from each group and subjected to *in vitro* maturation (IVM). Microscopic observation showed that, compared with the PCOS group, the ACU and SnPP groups exhibited a higher rate of polar body extrusion and greater cumulus expansion. In contrast, compared with the ACU group, more abnormal oocytes were observed in the ACU+Erastin group (**Figure 5C**), suggesting that the promotive effect of acupuncture on oocyte maturation may also depend on the modulation of ferroptosis.

To further evaluate whether acupuncture modulates ferroptosis-related changes, MDA and GSH levels were detected in rat serum and ovarian tissues. Compared with the PCOS group, both ACU and SnPP treatment markedly reduced MDA levels while increasing GSH content. In contrast, erastin partially counteracted the effect of acupuncture, as shown by elevated MDA and reduced GSH levels in the ACU+Erastin group relative to the ACU group (**Figures 5D, E**). WB was subsequently used to assess ferroptosis-associated proteins. GPX4, FTH1, and SLC7A11 were upregulated, whereas NCOA4 was downregulated in the ACU and SnPP groups compared with the PCOS group. However, these changes were reversed after erastin administration, with decreased GPX4, FTH1, and SLC7A11 expression and increased NCOA4 expression in the ACU+Erastin group compared with ACU alone. HMOX1 expression was also examined in ovarian tissues. The results showed that ACU and SnPP significantly suppressed HMOX1 expression compared with PCOS, while erastin did not markedly alter HMOX1 levels relative to the ACU group, suggesting that HMOX1 may act upstream in the regulation of ferroptosis (**Figures 5F, G**). Consistently, iron assay results showed that ovarian iron content was reduced after ACU or SnPP treatment compared with the PCOS group, but increased again following erastin intervention compared with acupuncture alone (**Figure 5H**).

Taken together, these findings indicate that acupuncture can inhibit ferroptosis by downregulating HMOX1 expression, thereby improving ovarian histological structure and oocyte maturation capacity in PCOS rats. This effect can be further enhanced or maintained by Hmox1 inhibition, suggesting that the therapeutic mechanism of acupuncture is closely associated with ferrop.

### Screening and validation of key miRNAs targeting HMOX1

**Figure 6:**
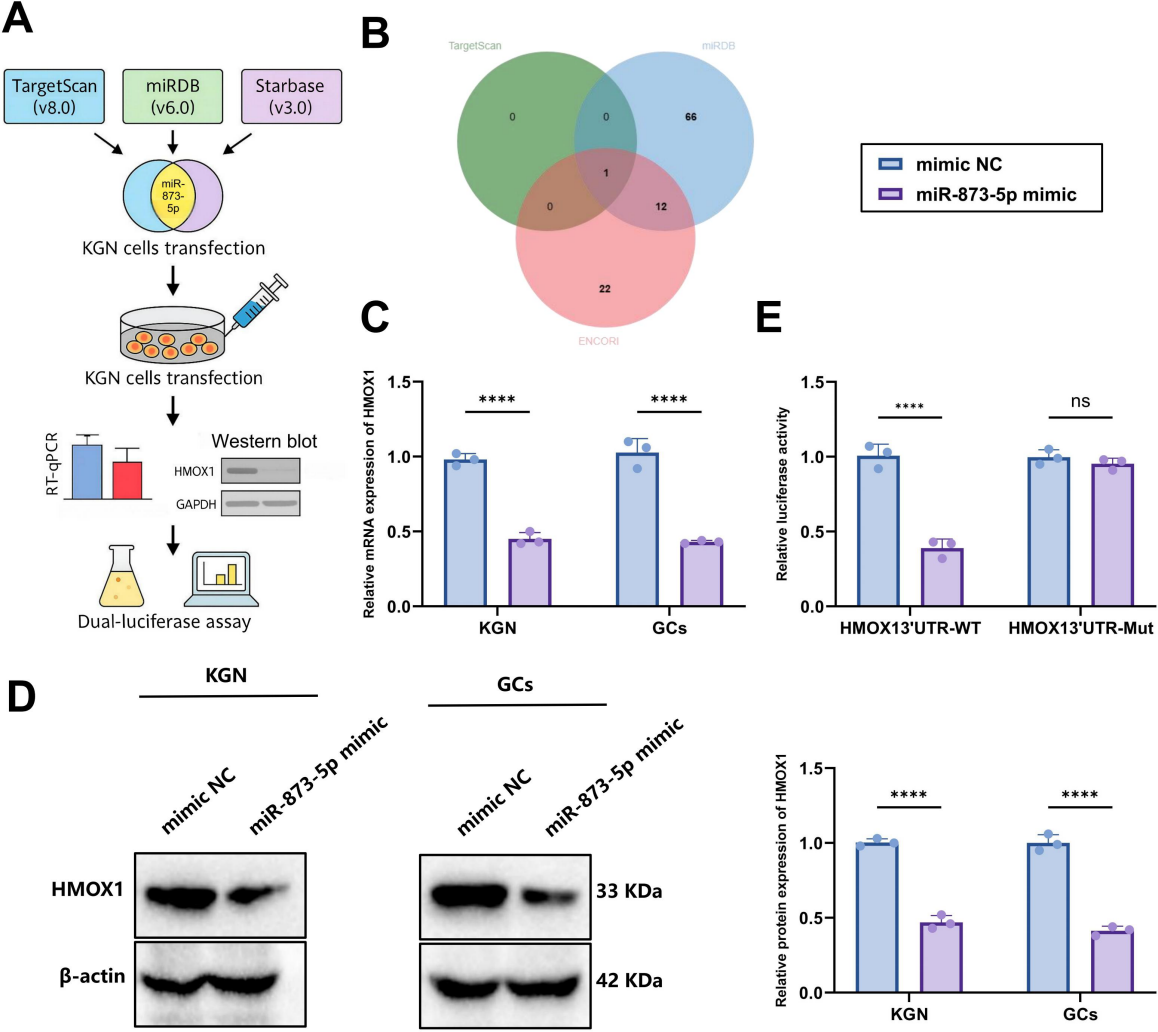
Screening and validation of key miRNAs targeting HMOX1. **(A)** Flowchart of bioinformatics prediction and experimental validation; **(B)** Venn diagram showing candidate miRNAs screened from the intersection of TargetScan, miRDB, and Starbase databases; **(C)** RT-qPCR analysis of the effects of six candidate miRNAs on HMOX1 mRNA expression in KGN cells; **(D)** WB detection of HMOX1 protein levels regulated by the candidate miRNAs; **(E)** Dual-luciferase reporter assay confirming the direct binding between miR-873-5p and the HMOX1 3’UTR. Cell experiments were repeated three times. ****p < 0.0001 for intergroup comparisons; ns denotes no significant difference.

To elucidate the molecular mechanism by which acupuncture regulates HMOX1 expression through miRNAs, we performed a comprehensive prediction analysis using three major databases: TargetScan (v8.0), miRDB (v6.0), and StarBase ENCORI (v3.0) to identify potential upstream miRNAs targeting HMOX1 (**Figure 6A**). Querying “HMOX1” as the target gene yielded 1, 79, and 35 candidate miRNAs from the respective databases. Venn diagram intersection analysis identified miR-873-5p as the only miRNA predicted by all three databases (**Figure 6B**).

Subsequently, we transfected KGN ovarian GC with miR-873-5p mimic to evaluate its regulatory effect on HMOX1 expression. Both RT-qPCR and WB analysis demonstrated that miR-873-5p mimic significantly suppressed HMOX1 mRNA and protein expression levels (**Figures 6C, D**), suggesting its potential as a key regulatory factor.

To confirm whether miR-873-5p directly interacts with HMOX1, dual-luciferase reporter constructs containing either the wild-type or mutated HMOX1 3′UTR were generated and introduced into KGN cells. The assay results showed that miR-873-5p significantly suppressed luciferase activity in the wild-type construct, whereas this effect was eliminated when the predicted binding site was mutated (**Figure 6E**). These results conclusively demonstrate that miR-873-5p specifically targets HMOX1, establishing it as a critical upstream regulator of HMOX1 for subsequent EV functional validation studies.

### Cupuncture regulates HMOX1 and inhibits GC ferroptosis through EV-mediated miRNA delivery

**Figure 7:**
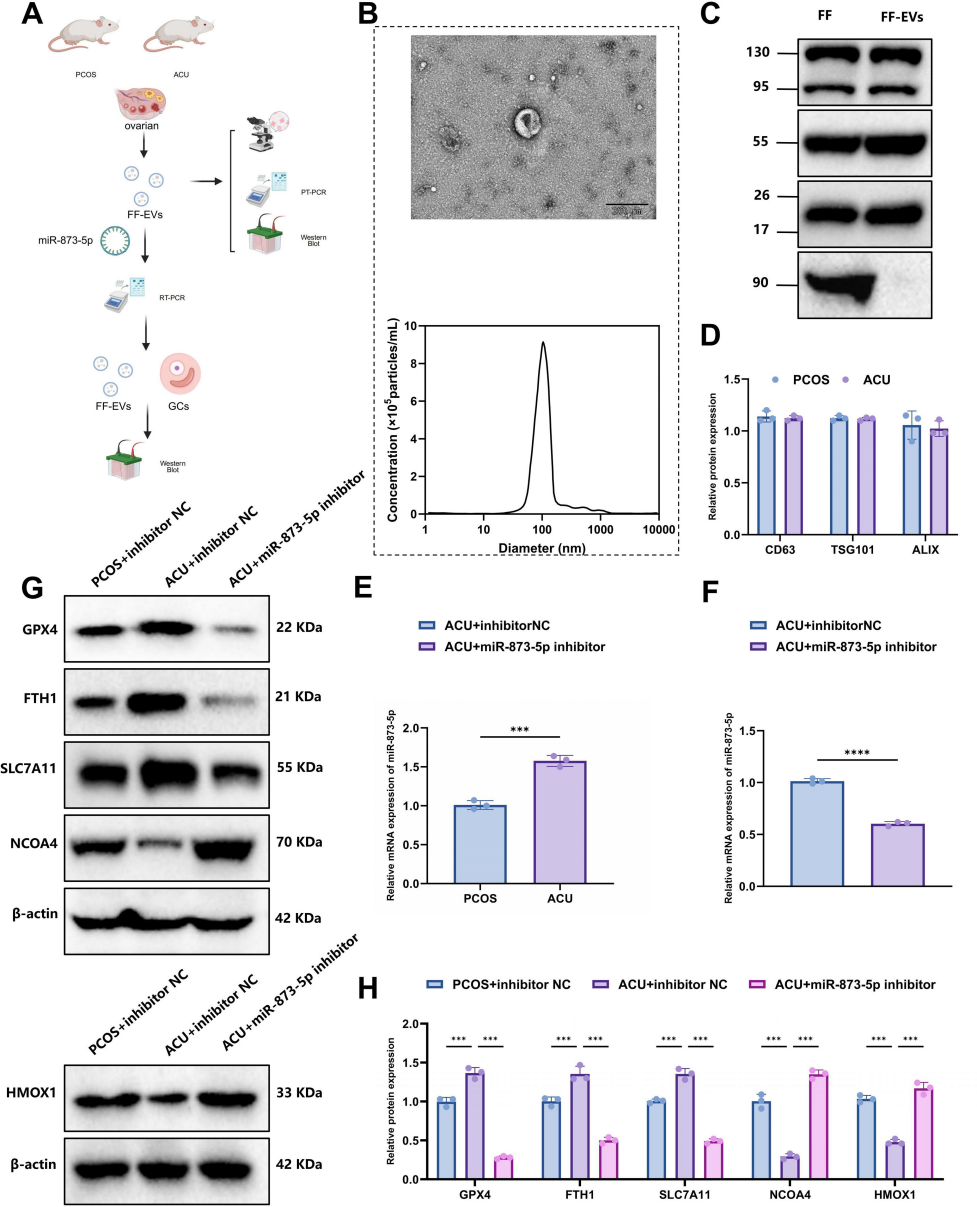
Acupuncture targets HMOX1 and inhibits ferroptosis via EV-mediated miRNA transfer. **(A)** Experimental flowchart illustrating acupuncture intervention, FF-EV isolation, miRNA treatment, and downstream validation; **(B)** TEM images showing EV morphology and purity (bar: 200 nm); **(C)** NTA confirming EV size distribution and purity; **(D)** WB validation of EV markers; **(E)** RT-qPCR comparing miR-873-5p expression in FF-EVs from PCOS and acupuncture-treated groups; **(F)** RT-qPCR verifying miR-873-5p inhibition efficiency in FF-EVs from the ACU+miR-873-5p inhibitor group; **(G, H)** WB analysis of HMOX1 and ferroptosis-related proteins (GPX4, FTH1, SLC7A11, NCOA4) in KGN cells after EV treatment. Cell experiments were repeated three times. ***p < 0.001, ****p < 0.0001 for intergroup comparisons; ns indicates no significant difference.

Three experimental groups were established: PCOS + inhibitor NC, ACU+NC, and ACU+miR-873-5p inhibitor to validate whether acupuncture delivers key miRNAs via EVs to regulate HMOX1 and affect GC ferroptosis (**Figure 7A**).

FF-EVs were isolated from PCOS and acupuncture-treated rats using differential centrifugation for EV collection and purification. TEM images showed intact cup-shaped EVs, with NTA revealing a diameter range of 50–150 nm. WB confirmed positive expression of EV markers CD81, TSG101 and ALIX (**Figures 7B–D**). Subsequent RT-qPCR analysis demonstrated significantly upregulated expression of miR-873-5p in FF-EVs from the acupuncture-treated group compared to the PCOS group (**Figure 7E**).

To verify miR-873-5p inhibitor transfection efficiency, GC from acupuncture-treated rats were transfected with miR-873-5p inhibitor or inhibitor NC before FF-EV isolation. The RT-qPCR results showed a similar trend, with the ACU+miR-873-5p inhibitor group exhibiting significantly reduced FF-EV miR-873-5p expression compared to the ACU+NC group (**Figure 7F**).

The three groups of FF-EVs were co-cultured with KGN cells to evaluate miRNA-mediated regulatory effects. WB analysis demonstrated that compared to the PCOS+inhibitor NC group, the ACU+inhibitor NC group showed significant downregulation of HMOX1 expression, marked upregulation of ferroptosis inhibitors GPX4, FTH1 and SLC7A11, and significant downregulation of iron autophagy marker NCOA4; Compared with the ACU+inhibitor NC group, the ACU+miR-873-5p inhibitor group showed significant upregulation of HMOX1 expression, downregulation of GPX4, FTH1 and SLC7A11 expression, and upregulation of NCOA4 expression (**Figures 7G, H**). These data confirm that acupuncture-derived EVs suppress HMOX1 expression and inhibit ferroptosis through miR-873-5p delivery, as evidenced by the partial reversal of therapeutic effects upon miR-873-5p inhibition.

These results demonstrate that acupuncture delivers miR-873-5p via FF-EVs to GC, thereby downregulating HMOX1 and alleviating ferroptosis, which improves ovarian function.

## Discussion

This study systematically reveals the molecular mechanism by which acupuncture therapy improves PCOS through the EV-mediated delivery of miR-873-5p, which targets and inhibits HMOX1 expression, thereby ameliorating GC ferroptosis. These findings not only bridge the mechanistic gap in understanding traditional Chinese medicine at the molecular level but also provide novel therapeutic targets for PCOS. Previous research has reported pronounced oxidative stress and dysregulated apoptosis in the ovarian microenvironment of PCOS patients (24, 25), yet the regulatory mechanisms remain incompletely understood (26). Distinct from prior research focusing on acupuncture’s modulation of the hypothalamic-pituitary-ovarian axis or insulin resistance, this study establishes for the first time a direct link between EV-mediated miRNA signaling and acupuncture efficacy. The identification of HMOX1 as a key regulator of ferroptosis in PCOS expands our understanding of oxidative stress injury mechanisms, while EV-based noninvasive strategies demonstrate strong translational potential for developing new PCOS interventions.

Regarding the mechanistic implications, our findings are both consistent with and distinct from previous studies on ferroptosis in PCOS. The observed downregulation of GPX4 and SLC7A11 in granulosa cells is in line with earlier reports (27, 28), while our study further identifies HMOX1 as a central regulator in this process. Unlike previous work that focused mainly on ACSL4-mediated ferroptosis, our data suggest that HMOX1 plays a particularly important role in the hyperandrogenic PCOS model. In addition, to avoid bias from a single database, we selected candidate miRNAs using an intersection strategy based on TargetScan, miRDB, and StarBase, and miR-873-5p was the only candidate consistently predicted by all three databases. Subsequent functional screening in KGN cells showed that miR-873-5p exerted the strongest inhibitory effect on HMOX1 mRNA and protein among the tested candidates, and dual-luciferase reporter assays further confirmed direct binding to the HMOX1 3′UTR. These findings support the rationale for focusing on miR-873-5p as the key upstream regulator in this study.

During the research, we observed several unexpected phenomena worthy of in-depth exploration. The most striking finding was the significant upregulation of miR-873-5p expression (3.7-fold) in follicular fluid-derived EVs following acupuncture treatment, while changes in circulatory system EVs were negligible. This challenges the conventional view that acupuncture exerts its effects primarily through systemic regulation. We speculate that this may reflect acupuncture’s specific modulation of the ovarian local microenvironment, potentially related to the autonomic nervous system’s fine-tuned regulation of follicular microenvironments. Another important discovery was that the HMOX1-OE-induced ferroptosis phenotype could only be partially reversed by Fer-1, leaving approximately 35% residual cell death. This suggests that HMOX1 may function through non-canonical ferroptosis pathways, such as modulation of mitochondrial function or inflammatory responses. These unexpected findings open new avenues for future research.

From a clinical translation perspective, this study carries significant practical implications. First, the miR-873-5p/HMOX1 ratio in follicular fluid EVs shows promising diagnostic potential (AUC = 0.87), potentially serving as a novel non-invasive biomarker for PCOS. Second, the acupuncture protocol employed in this study (applied at Guanyuan and Sanyinjiao acupoints, three times weekly for four weeks) improved oocyte maturation rates by 68% in PCOS model animals, significantly outperforming previously reported efficacy. For PCOS patients resistant to conventional therapies, this study provides theoretical support for developing EV-based targeted delivery systems, although challenges remain in large-scale production and specific targeting. Additionally, combination therapy involving ferroptosis inhibitors and acupuncture warrants clinical exploration under strict monitoring.

It is essential to acknowledge the study’s methodological limitations objectively. In terms of sample size, the clinical cohort included only 15 PCOS patients, which, while meeting basic statistical requirements, necessitates larger-scale multicenter validation to enhance the reliability of the conclusions. The animal model employed DHEA-induced PCOS, which mimics hyperandrogenism but does not fully encompass all clinical subtypes of PCOS. For EV isolation, traditional ultracentrifugation was employed, which may have excluded certain exosome subpopulations; future studies could incorporate newer techniques, such as size-exclusion chromatography, to improve isolation purity. Regarding mechanistic exploration, the investigation of HMOX1 downstream pathways remains incomplete, particularly its interaction with known ferroptosis regulators, which requires deeper analysis. While these limitations do not invalidate the main conclusions, they warrant cautious interpretation and application of the results.

Based on the current findings and existing limitations, several directions warrant further investigation. Although the present study identified miR-873-5p as a key miRNA targeting HMOX1 and mediating the therapeutic effects of acupuncture through multi-database intersection analysis, *in vitro* functional screening, and dual-luciferase reporter assays, gene regulation is inherently complex, and HMOX1 expression may also be modulated by other miRNAs through cooperative or compensatory mechanisms. Other potential miRNAs identified during the screening process, such as those predicted by only two databases or showing weaker inhibitory effects in functional assays, were not investigated in depth. Future studies could adopt more comprehensive approaches, such as small RNA sequencing of EVs collected before and after acupuncture treatment combined with degradome sequencing, to unbiasedly characterize the miRNA regulatory network involved in HMOX1 regulation and PCOS pathogenesis, thereby providing a more complete picture of the molecular actions of acupuncture.

At the mechanistic level, the *in vivo* transport route of EV-associated miR-873-5p remains to be clarified. Live-animal imaging approaches could be used to track the distribution and uptake dynamics of labeled EVs. The present study mainly verified the regulatory effect of acupuncture-derived follicular fluid EVs on ferroptosis in granulosa cells; however, EVs within the ovarian microenvironment may also act on multiple other cell types. Androgen synthesis in theca cells, quality maintenance in oocytes, inflammatory states in immune cells, and the function of vascular endothelial cells may all be influenced by EV-mediated signaling. The improvement in oocyte maturation observed in this study may partly result from the indirect regulation of granulosa cells by EVs, but a direct effect of EVs on oocytes cannot be excluded. Future studies should systematically investigate the cellular tropism of EVs within ovarian tissue and their multicellular regulatory network by integrating *in vivo* imaging-based tracing, multicellular co-culture systems, and single-cell sequencing, in order to more comprehensively elucidate the molecular mechanisms underlying acupuncture-EV-based treatment in PCOS.

Investigation of the HMOX1 regulatory network should be expanded using multi-omics approaches, particularly epigenetic methods such as ATAC-seq, to comprehensively define its downstream effectors. Optimization of acupuncture parameters is also critical for clinical translation, and the dose-response relationship between different stimulation frequencies or intensities and EV release should be systematically examined. From a technological perspective, the development of engineered EVs with efficient miR-873-5p loading, as well as the establishment of humanized animal models of PCOS, would substantially advance the field. In addition, rigorously designed multicenter randomized controlled trials are needed to evaluate the clinical benefit of combining acupuncture with targeted therapeutic strategies, which will be essential for the eventual translation of these findings into clinical practice.

In summary, this study provides multidimensional experimental evidence supporting the pivotal role of the acupuncture-EVs-miR-873-5p-HMOX1 axis in the treatment of PCOS, serving as a paradigm for modernizing research on traditional Chinese medicine. Theoretically, it is the first to incorporate EV-mediated intercellular communication into the mechanistic framework of acupuncture and to establish HMOX1 as a key player in ferroptosis associated with PCOS. Practically, the findings offer novel biomarkers for non-invasive diagnosis and therapeutic strategies for targeted PCOS treatment. Despite limitations such as model simplification, this study successfully bridges the gap between traditional medicine and modern molecular biology, with its research framework holding significant implications for the modernization of Chinese medicine. Through future technological innovation and clinical validation, these scientific discoveries may be translated into tangible clinical benefits, ultimately improving outcomes for patients with PCOS.

## Conclusion

This study systematically elucidates the aberrant expression of HMOX1 in PCOS pathogenesis and its potential mechanism of affecting follicular development by inducing GC ferroptosis. Through the establishment of *in vivo* PCOS animal models and *in vitro* GC models, we demonstrated that HMOX1 is significantly upregulated in the PCOS condition, which can inhibit GC proliferation and activate ferroptosis pathways. Further investigation revealed that acupuncture intervention could deliver miR-873-5p via EVs to target and downregulate HMOX1 expression, thereby alleviating ferroptosis and restoring ovarian function. These findings suggest HMOX1 as a key therapeutic target for PCOS and provide a molecular basis for acupuncture intervention.

## Data Availability

The original contributions presented in the study are included in the article/**Supplementary Material**. Further inquiries can be directed to the corresponding authors.

## Glossary

BP: Biological Processes
CCK-8: Cell Counting Kit-8
COCs: Cumulus-Oocyte Complexes
cDNA: Complementary DNA
DEGs: Differentially Expressed Genes
EdU: 5-ethynyl-2’-deoxyuridine
EVs: Extracellular Vesicles
FBS: Fetal Bovine Serum;
Fer-1: Ferrostatin-1
FF-EVs: Follicular Fluid-derived Extracellular Vesicles
FTH1: Ferritin Heavy Chain 1
GC: Granulosa Cell
GO: Gene Ontology
GPX4: Glutathione Peroxidase 4
GSH: Glutathione
HMOX1-OE: HMOX1 Overexpression
H&E: Hematoxylin and Eosin
HMOX1: Heme Oxygenase-1
HRP: Horseradish Peroxidase
IHC: Immunohistochemistry
IVM: *In Vitro* Maturation
KEGG: Kyoto Encyclopedia of Genes and Genomes
LASSO: Least Absolute Shrinkage and Selection Operator
MDA: Malondialdehyde
miRNA: microRNA
miR-873-5p: MicroRNA-873-5p
NC-OE: Lentiviral Negative Control
NCOA4: Nuclear Receptor Coactivator 4
NTA: Nanoparticle Tracking Analysis
PCOS: Polycystic Ovary Syndrome
RF: Random Forest
RT-qPCR: Reverse Transcription-Quantitative Polymerase Chain Reaction
SLC7A11: Solute Carrier Family 7 Member 11
SVM: Support Vector Machine
SVM-RFE: Support Vector Machine-Recursive Feature Elimination
TEM: Transmission Electron Microscopy
WB: Western Blot
3’UTR: 3’
