# Supplementary material for: Acupuncture modulates extracellular vesicle-miRNA-HMOX1 axis to inhibit ferroptosis in polycystic ovary syndrome: integrating machine learning, in vitro and in vivo studies
Source: Front Endocrinol (Lausanne). 2026 Jul 23;17:1790049. doi: 10.3389/fendo.2026.1790049 (PMC13441814; doi:10.3389/fendo.2026.1790049)
Supplement: Supplementary file 1 [file Table1.docx]

**Table S1. RT-qPCR primer sequences.**

| **Gene** | **Primer Sequence** |
| --- | --- |
| GADPH (rat) | F: 5'-CACCATCTTCCAGGAGCGAG-3' |
|  | R: 5'-GGCGGAGATGATGACCCTTT-3' |
| GADPH (human) | F: 5'-AATGGGCAGCCGTTAGGAAA-3' |
|  | R: 5'- GCGCCCAATACGACCAAATC-3' |
| HMOX1 (rat) | F: 5'-TGCACATCCGTGCAGAGAAT-3' |
|  | R: 5'-CTGGGTTCTGCTTGTTTCGC-3' |
| HMOX1 (human) | F: 5'-AAGACTGCGTTCCTGCTCAAC-3' |
|  | R: 5'-AAAGCCCTACAGCAACTGTCG-3' |

Note: F: Forward, R: Reverse.

**Table S2. Details of the first antibody product.**

| **Name** | **Cat.** | **DiIution ratio** | **Manufacturer** | **Country** | **MW (kDa)** |
| --- | --- | --- | --- | --- | --- |
| HMOX1 | ab189491 | 1: 2000 | Abcam | UK | 33 |
| GPX4 | ab125066 | 1: 1000 | Abcam | UK | 22 |
| FTH1 | ab75973 | 1: 1000 | Abcam | UK | 21 |
| CD81 | ab109201 | 1: 1000 | Abcam | UK | 26 |
| TSG101 | ab125011 | 1: 1000 | Abcam | UK | 44 |
| ALIX | ab275377 | 1: 1000 | Abcam | UK | 95 |
| SLC7A11 | ab307601 | 1: 1000 | Abcam | UK | 55 |
| NCOA4 | ab314553 | 1: 1000 | Abcam | UK | 70 |
| calnexin | ab13504 | 1: 2000 | Abcam | UK | 90 |
| β-actin | ab8227 | 1: 1000 | Abcam | UK | 42 |
